# Grid cells on steeply sloping terrain: evidence for planar rather than volumetric encoding

**DOI:** 10.3389/fpsyg.2015.00925

**Published:** 2015-07-15

**Authors:** Robin M. A. Hayman, Giulio Casali, Jonathan J. Wilson, Kate J. Jeffery

**Affiliations:** ^1^Department of Clinical and Experimental Epilepsy, Institute of Neurology, Faculty of Brain Sciences, University College LondonLondon, UK; ^2^Institute of Behavioural Neuroscience, Research Department of Experimental Psychology, Division of Psychology and Language Sciences, University College LondonLondon, UK

**Keywords:** spatial cognition, navigation, place cells, grid cells, theoretical model, dimensions

## Abstract

Neural encoding of navigable space involves a network of structures centered on the hippocampus, whose neurons –place cells – encode current location. Input to the place cells includes afferents from the entorhinal cortex, which contains grid cells. These are neurons expressing spatially localized activity patches, or firing fields, that are evenly spaced across the floor in a hexagonal close-packed array called a grid. It is thought that grids function to enable the calculation of distances. The question arises as to whether this odometry process operates in three dimensions, and so we queried whether grids permeate three-dimensional (3D) space – that is, form a lattice – or whether they simply follow the environment surface. If grids form a 3D lattice then this lattice would ordinarily be aligned horizontally (to explain the usual hexagonal pattern observed). A tilted floor would transect several layers of this putative lattice, resulting in interruption of the hexagonal pattern. We model this prediction with simulated grid lattices, and show that the firing of a grid cell on a 40°-tilted surface should cover proportionally less of the surface, with smaller field size, fewer fields, and reduced hexagonal symmetry. However, recording of real grid cells as animals foraged on a 40°-tilted surface found that firing of grid cells was almost indistinguishable, in pattern or rate, from that on the horizontal surface, with if anything increased coverage and field number, and preserved field size. It thus appears unlikely that the sloping surface transected a lattice. However, grid cells on the slope displayed slightly degraded firing patterns, with reduced coherence and slightly reduced symmetry. These findings collectively suggest that the grid cell component of the metric representation of space is not fixed in absolute 3D space but is influenced both by the surface the animal is on and by the relationship of this surface to the horizontal, supporting the hypothesis that the neural map of space is “multi-planar” rather than fully volumetric.

## Introduction

**FIGURE 1:**
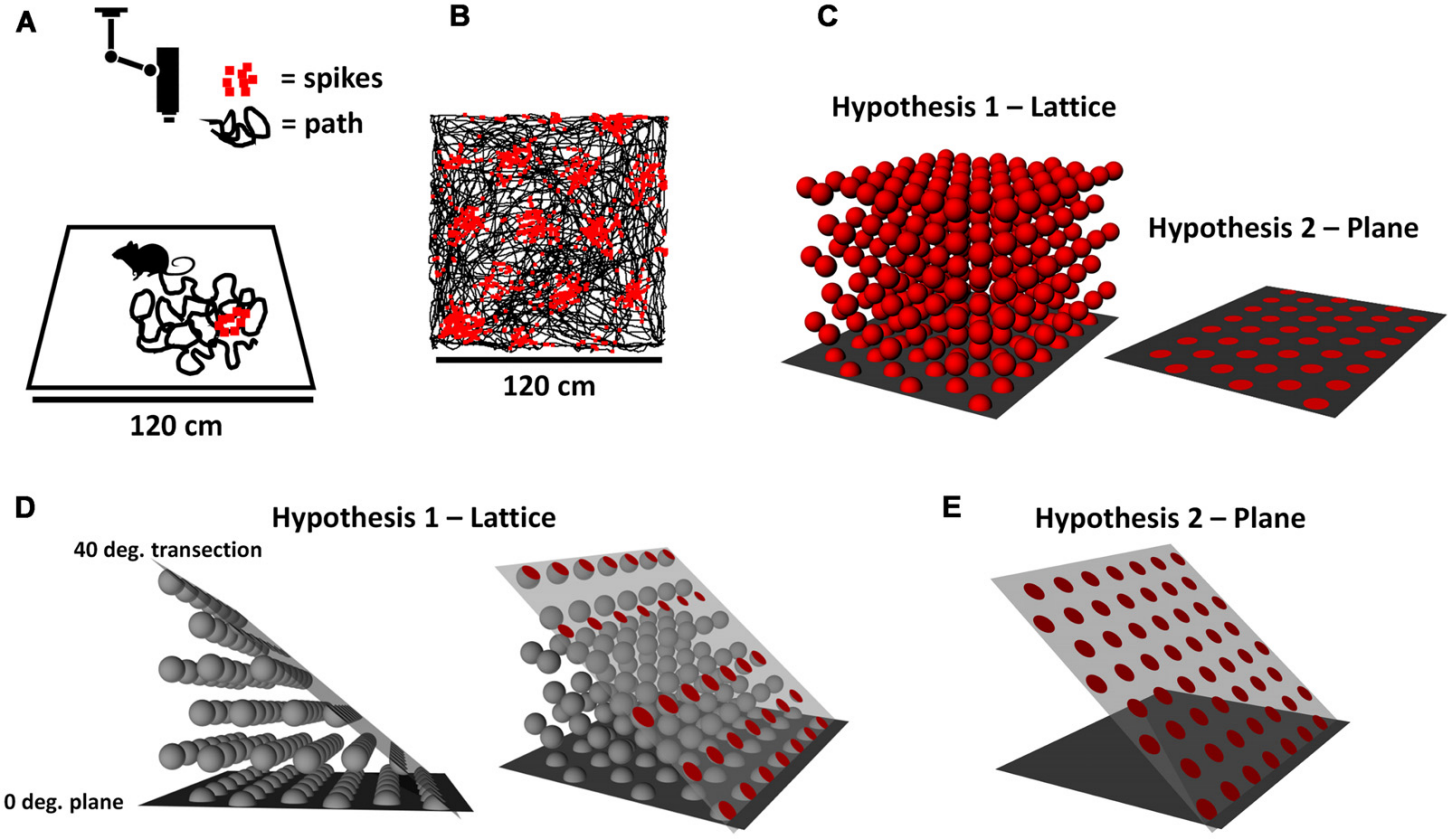
**(A)** Neurons recorded from a rat foraging on a square arena, monitored by an overhead video camera. The black lines represent the recent path of the meandering rat, and the red squares represent spikes emitted by an entorhinal grid cell. **(B)** Raw data from a real grid cell, showing how the spikes are laid down in regularly spaced clumps (“fields”), forming a close-packed hexagonal array across the surface of the environment. **(C)** Two hypotheses about the structure of grids in three-dimensional (3D) space: that they are a lattice that permeates the space (left), or a plane that lies only on the environment surface (right). **(D)** Two views (for ease of visualization) of how a lattice grid would intersect a surface sloped at 40° from horizontal. The red circles on the right show the surfaces of grid fields that intersect the surface. **(E)** If the grid is instead planar and follows the tilted surface, the fields would retain the hexagonal close-packed arrangement they had on the horizontal plane. Notice the increased number of fields and packing density, and their more symmetrical arrangement.

Place cells in the hippocampus of freely foraging mice, rats, and humans emit action potentials (spikes) when the organism is in a specific location in space, and are thought to provide the neural basis for the sense of self-location (Moser et al., 2008). Recordings of neurons in posterior cortical areas [including the medial entorhinal cortex (MEC) and pre- and para-subiculum(PaS)], made as an animal explores a large arena (**Figure 1A**), find that the cells fire in an evenly spaced array of locations organized into a regular hexagonal close-packed array (**Figure 1B**) known as a grid (Hafting et al., 2005). It is thought that this spatially regular organization, and in particular the relatively constant inter-field distances, may allow the calculation of distances and directions to aid the spatial computations supporting place cell activity.

Most animals do not in fact move around on a completely flat, two-dimensional (2D) surface, but rather explore a more complex environment having hills, valleys, tunnels, branches, etc. The animals are therefore not constrained to moving only in the two dimensions of the horizontal plane, but have a component of their movement that goes vertically, into the third (vertical) dimension (see our companion review paper, Jeffery et al., 2015, for a fuller discussion of these issues). Indeed, many animals that can fly or swim (including our aquatic ancestors) move freely in three-dimensional (3D) spaces without any constraints – that is, in all three dimensions, or in what we call here *volumetric space*. This raises the question of how the grid cells might encode 3D space. We consider here two competing hypotheses (**Figure 1C**). The first is that the array of grid fields that is sampled by animals moving around on a horizontal surface actually extends into volumetric space – that is, grid “fields” would actually be spherical and be distributed throughout the space if we could record while an animal sampled the space freely (as birds, fish, and bats do). This proposition has been examined in theoretical work that has predicted that fields would likely be distributed in a close-packed 3D lattice (Horiuchi and Moss, 2015; Mathis et al., 2015), of which the two most efficient forms (described below) are hexagonal close-packed and face-centered cubic. Alternatively, it may be that grid fields follow the surface of the environment and thus maintain the same metric characteristics that they do on the horizontal plane. These two scenarios have different implications for how animals might calculate distances across non-flat terrain.

We investigated these hypotheses with a combination of modeling and grid cell recording. For the modeling, we determined the predicted pattern of grid cell firing if the underlying structure of grid fields is a lattice extending into volumetric space (**Figure 1D**), vs. if it is planar and follows the surface of the environment (**Figure 1E**). A lattice is the arrangement that would occur if grid fields were spheres that had been packed together into a box; the closest arrangement of such spheres forms a volumetric close-packed array. For a purely planar packing, where the fields are disks instead of spheres and packed together on a surface instead of within a space, then the arrangement is a planar close-packed array, in which the disks form themselves into a hexagonal pattern. Note that if a volumetric close-packed array is aligned to the horizontal plane (as it would be for, say, a box of oranges settling under the influence of gravity) then the arrangement of the bottom layer of the lattice is a planar close-packed array. Since grid cell firing patterns do in fact form a planar close-packed array, the question we asked is – is this because their encoding is purely planar, or is it because it is volumetric but only the bottom layer of fields is sampled in a typical experiment?

**FIGURE 2:**
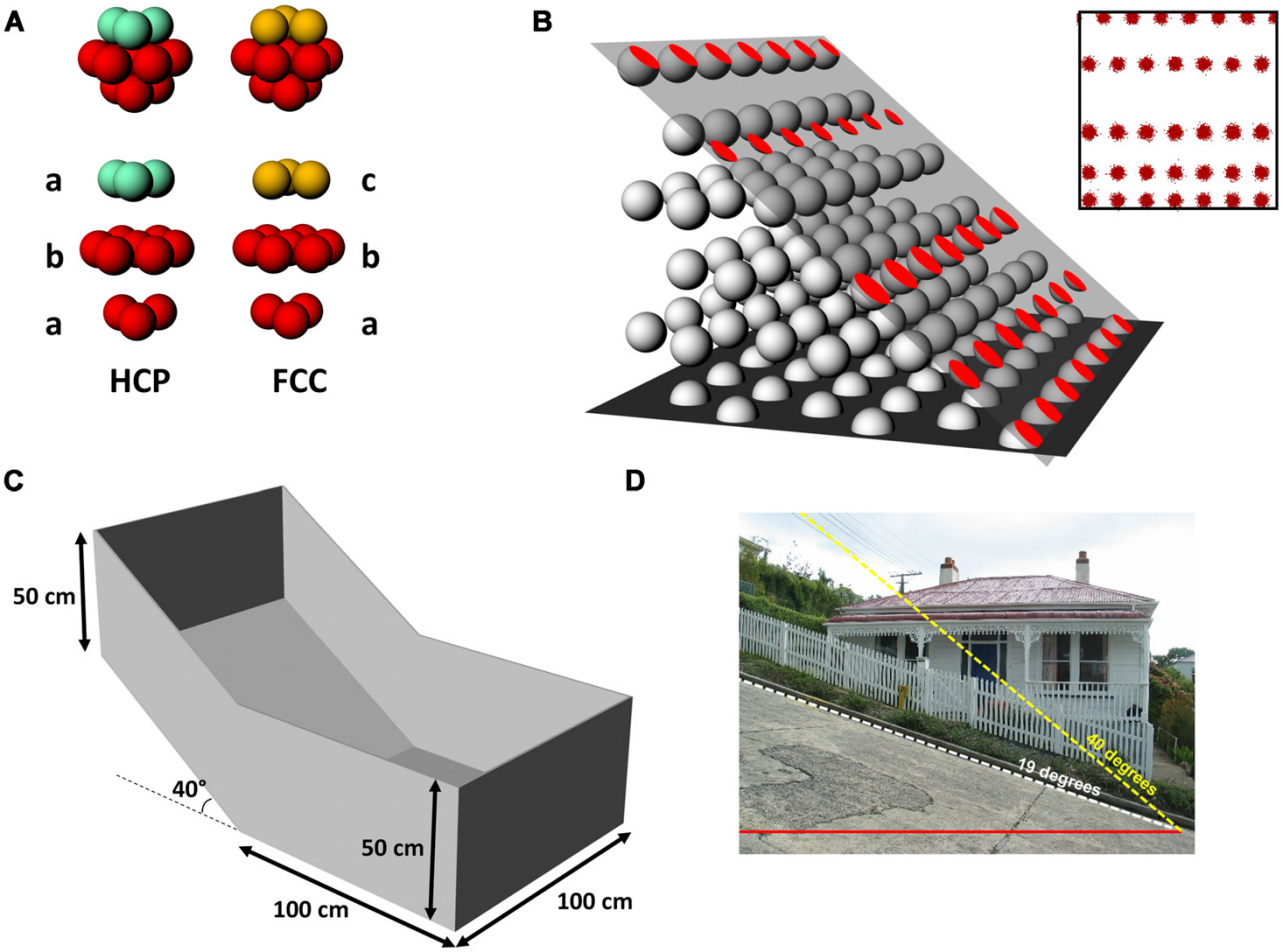
**Basic methodology for the study, showing the model generation and interrogation and the recording apparatus. (A)** The two forms of 3D closest packing used in our simulation: hexagonal close-packed (HCP), which comprises alternating layers of differing horizontal offsets (ababab…), and face-centerd cubic (FCC) for which there are three layers (abcabc…). The layers that do not differ are shown in red; turquoise and gold indicate the layers that distinguish the packings. **(B)** Generation of the spike plots. An imaginary plane was placed into the lattice at a given orientation and offset, creating a set of circles where the plane intersected the spheres in the lattice. The parameters derived from **(B)** were then used to generate simulated spikeplots (inset) which were then analyzed in the same way as for real spikes. **(C)** Schematic of the recording box used for grid cell data collection, with one half flat and one half tilted 40°. **(D)** For reference, 40° is slightly over twice the steepness of the “world’s steepest street,” Baldwin Street, Dunedin, NZ. “Baldwinstreet”. Photgraph licensed under Public Domain via Wikimedia Commons – http://commons.wikimedia.org/wiki/File:Baldwinstreet.jpg#/media/File:Baldwinstreet.jpg.

For the volumetric hypothesis we assumed that if the intrinsic grid pattern in 3D space *is* a lattice, it would be aligned to the horizontal plane, which would explain why the usual pattern seen is a close-packed hexagonal array (the maximally efficient packing pattern). We thus generated horizontally aligned close-packed lattices [of which there are two forms, hexagonal close packed (HCP) and face centered cubic (FCC), which are shown in **Figure 2A** and described below], and then determined what would happen to the grid pattern assuming an animal walked on a steep slope that cut through the grids (**Figure 2B**).

For the recording, we created a two-compartment arena in which one half was a 1 m × 1 m horizontal square and the other half was a square of the same size, tilted at 40° (**Figure 2C**). Rats were implanted with electrodes aimed at MEC. When grid cells were isolated, the rats were allowed to forage over the entire arena while recordings of the grid pattern were made. We then compared the statistics of the firing patterns with the predictions made by the model. We show here that in the model, transections of a grid lattice for all angles other than horizontal result in a decrease in the area covered by the activity, with one exception which is the FCC pattern cut at 70°. Our recordings showed, however, in fact a slight increase in both coverage and field number, a finding that is more consistent with the grid cell grid having followed the tilted surface (in a slightly degraded way), rather than being a volumetric lattice that was cut through at an angle. However, there was a decrease in the coherence and symmetry of the firing patterns, suggesting that locomoting on a steep slope did have some effect on grid cell firing. We discuss the implications of these findings for how rats may encode 3D space, and explore the issues more fully in the companion paper (Jeffery et al., 2015).

## Materials and Methods

We describe first the modeling, and then the single neuron recording protocol.

### Three-Dimensional Model of Grid Cells

A model of grid firing in three-dimensions was constructed, in order to interpret the patterns of actual grid cell firing observed in the single neuron recording study. In two dimensions grid fields optimally fill the plane in a close-packed hexagonal tiling (Gauss, 1831), which is the most efficient way to pack circles on a plane (note that for grid fields there are spaces between the fields – one can therefore think of a grid field as comprising a patch of spiking surrounded by an empty annulus of the same thickness as the field radius). In 3D space, there are many different packing arrangements of equally sized spheres but only two maximally efficient ones; HCP and FCC (**Figure 2A**). These arrangements are similar in that they both comprise stacked layers of spheres that have been packed into a close-packed hexagonal arrangement; the difference between them is how the layers are arranged. Each layer is translated slightly with respect to the one underneath: for HCP there are two translational positions and the layers alternate ABABAB…, for FCC there are three, and the layers repeat ABCABC…

The model simulated these two packing arrangements and was used to investigate what would happen when a 2D plane intersected the volume of spheres. Full details of the model can be found in the Supplementary Methods, but the brief details are as follows, illustrated in **Figure 2**. A volume was packed with spheres, each comprising a central core of radius 12 units, surrounded by an empty shell also 12 units thick (thus resembling grid fields). The inter-field distance was thus 24 units. The packing arrangement was either HCP or FCC (**Figure 2A**), with the layers aligned horizontally (this was assumed as the default because a horizontal transection of this lattice would yield a planar close-packed hexagonal array, which is what is seen with real grid cells). We then created a plane of dimensions 100 × 100 units (i.e., 4–5 fields across), which was placed into the volume at various tilts (rotations around a horizontal axis), orientations (rotations around a vertical axis), and offsets (translations orthogonal to the intersection of the plane with the horizontal plane). Wherever the plane intersected a sphere, the circular area of intersection was filled with simulated spikes (**Figure 2B**) using a Gaussian distribution centered on the circle’s center and modulated by its radius. This was repeated for all of the spheres intersected by the plane resulting in a ‘spike plot’ which served as the basis for a 2D histogram, equivalent to a rate-map of position (*x* and *y*) vs. firing rate (*z*). These rate-maps were then used to calculate measures such as inter-field distance and grid field occupancy of the plane and for constructing the spatial autocorrelograms (SAC) which are the basis of the grid score used to assess grid field regularity. We then generated a systematic set of planar sections through both packings, which were analyzed in detail for various statistics (described below in the section Data Analysis).

### Surgical Procedures

For the single neuron recording experiment, four animals were implanted with 16-channel microdrives (Axona Ltd, UK) each carrying four sets of tetrodes, each tetrode comprising four 25 micron diameter platinum-iridium wires. The tetrodes were aimed at the dorsal MEC at the following coordinates; AP: 0.1–0.3 mm anterior to the transverse sinus, ML: 4.0–4.5 mm to lambda, DV: 1.5–2.0 mm with the electrodes angled 8–10° anteriorly in the AP plane. The animals also participated as subjects in another study evaluating the effects on grid cells of changing environmental context (Marozzi et al., 2015). All procedures were performed under UK Home Office license authority according to the Animals (Scientific Procedures) Act 1986. All recordings were carried out on an Axona dacqUSB recording system (Axona Ltd, UK). Animals recovered post-operatively for 7 days before screening for grid cells commenced.

### Recording Apparatus

The recording apparatus, called here the “gradient box,” (**Figure 2C**) was made from medium-density fibreboard (MDF) and comprised two adjoining square floors, each 100 cm × 100 cm, one of which was tilted around the transverse midline axis of the arena by 40° with respect to the horizontal. This tilt was selected as being the steepest slope that the rats would willingly, and without slipping, traverse for food, and is twice as steep as the so-called “steepest street in the world” (**Figure 2D**). The flat part of the environment was continuous with the sloped section with no physical barrier between the two. Henceforth, for simplicity we will refer to the two halves of the box as “compartments” even though they were continuous. The entire arena was surrounded by walls 50 cm high as measured directly vertically upward from the surface of the recording arena; the walls at the highest point were oriented vertically (rather than orthogonal to the sloping floor) so as not to obscure the camera view. The box was painted with white paint on both the walls and floor and situated within a well-lit recording room that contained the recording apparatus, computer, and shelving, all of which were visible from the floor of the box and hence able to act as orienting cues for the rat.

### Screening and Recording Procedure

Recording began at least a week after surgery. Rats were screened for cells in a separate room, by being placed in a large 120 cm × 120 cm black or white box with 60 cm high walls. Once grid cells were identified, based on observer-apparent spatially localized multi-focal firing patterns, the animal was carried through to the recording room and a trial was run in the gradient box. Sweetened, cooked rice was thrown into the box to encourage the animal to forage freely; the rice was slightly wet so that it would not roll down the sloping floor and accumulate at the join between the two compartments. The camera used to track the animal’s position was located directly above the center of the box. Both the camera and the recording arena were kept in a fixed position throughout the experiment so that the correct transformation could be applied to the positional data *post hoc* to allow a comparison between the two compartments (see Positional Data below). The trials varied in duration between 15 and 30 min depending on the experimenter’s judgment of how completely the animal had sampled the spatial extent of the gradient box (the experimenter could see the cumulative path of the animal but not spike occurrences, during recording). This was done to ensure homogeneous sampling of both sides of the box. That this was successful is shown in the path analysis results.

### Data Analysis

#### Model Analysis

Having generated the model, we then repeatedly sampled it using a square cutting plane of 4–5 fields wide, to produce a cut surface containing circles (from where the plane intersected spheres). To create a comprehensive sampling of the lattice space, we positioned the plane at a variety of angular and linear orientations with respect to the lattice, as follows. For a given position of the plane’s central point, the plane was positioned in the lattice at tilts between 0° (horizontal) and 180° (horizontal again) in 3° increments, and for every tilt, it was rotated in 3° increments for the full 360° around the vertical axis. The position of the plane’s central point was then translated along the *x*-axis (i.e., in the direction of a row of fields) by 1/6th of the inter-field distance, and the rotational sampling repeated. This entire procedure (rotations followed by translation) was repeated until a full cycle of the pattern (six repetitions) had been sampled.

The circles generated as a result of the plane’s intersection with the lattice (**Figure 2B**) were then used to generate “fields” of spikes (**Figure 2B**), by using the centers and radii of the circles to form the mean and width, respectively, of a 2D Gaussian probability distribution, from which values were then drawn and used to create the spike plots for each individual field. These were then analyzed in the same way as for the actual neuronal data. Details of this procedure are thus given in the section on neuronal data analysis, below.

#### Positional Data and Its LFP Correlates

**FIGURE 3:**
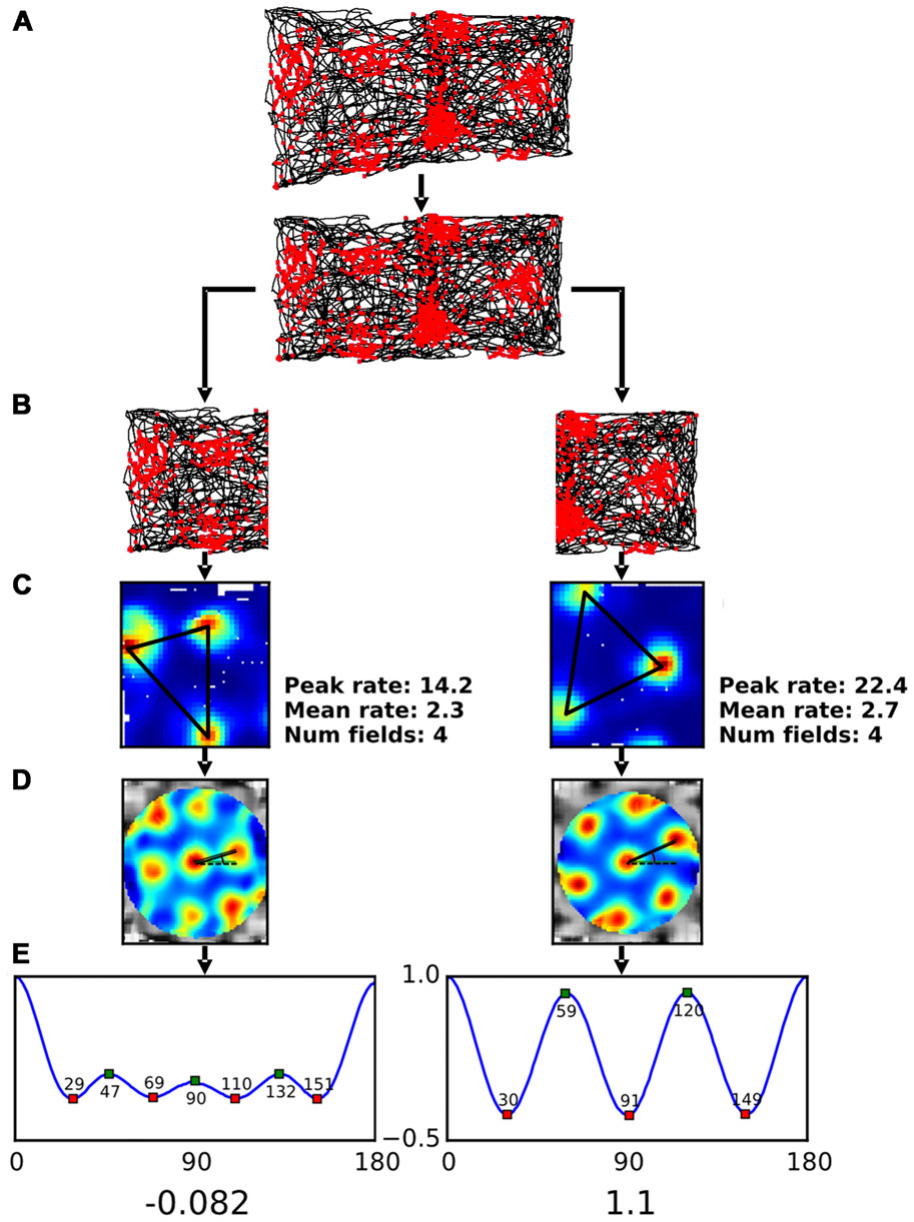
**Analysis procedure for single neuron data. (A)** A grid cell’s action potentials, or “spikes” (shown as waveforms seen on each of the four component electrodes of a tetrode) were plotted onto an image of the rat’s path as seen by an overhead camera (upper plot; spikes = red dots, path = black line). The resulting spikeplot was then rescaled (lower plot) to remove the effect of foreshortening produced by the 40°-tilt of the sloped (left) half of the apparatus. Note how the action potentials occurred in regularly spaced clusters, reflecting an odometric (distance-measuring) process. **(B)** The spikeplot was then divided into its sloped and flat components, and each analyzed independently. **(C)** The spikeplot was converted into a firing rate-map by binning the environment and creating a heat-map of firing rate per bin. The centers of each firing field were then determined and used for calculation of inter-field distances (black lines). **(D)** The rate-map was then converted into an autocorrelogram by successively translating it by small increments in *x*- and *y*-directions, and then correlating the shifted map with the original – where the translation resulted in one of the blobs mapping onto another the correlation was high (and likewise for the spaces between blobs). The resulting correlogram enhances the periodicity of the firing pattern. The black lines are drawn between the central blob and its two nearest neighbors – these values were used to calculate orientation (measured as angle from the dotted line). **(E)** In order to determine the symmetry of the autocorrelogram, a symmetry plot was generated, using an additional autocorrelation step in which the autocorrelogram was rotated in 1° increments and then correlated with the original – the resulting values are plotted as in the line graph, which shows the rising and falling correlation values as the autocorrelogram blobs come into and out of phase. For highly symmetrical hexagonal close-packed firing patterns, as occurs on the plot from the flat side, there are three peaks between 0° and 180° occurring one every 60°; for less symmetric patterns, as on the sloped side, the peaks are reduced in height and often (as in this example) changed in number. The green and red squares illustrate the locations of the peaks (values indicated); the values at the locations of expected troughs (30°, 90°, and 150°) are averaged and subtracted from the average values at the expected peaks (60° and 120°); the result is the grid score, shown by the numbers beneath the symmetry plots.

The camera that tracked the animals’ position was located over the x, y midpoint of the gradient box and captured the position of a single LED mounted on the head-stage at a sampling frequency of 50 Hz. Due to the design of the recording environment and the position of the camera the positional data captured on the sloped half of the gradient box appeared distorted (see **Figure 3A** for an example). In order to allow comparison with the data collected in the flat half of the environment it was necessary to correct this distortion using a transformation algorithm fully described in Supplementary Methods.

The transformed LED position values were smoothed with a 400 ms moving average filter and filtered to remove speeds in excess of 4 m/s (i.e., tracking errors). For the theta/speed analysis the speed filter was narrowed to only include speeds between 4 and 30 cm/s and for the remaining analyses the speed filter was expanded to include speeds between 5 and 60 cm/s.

The heading direction of the animals was calculated based on the current run direction as there was only one LED on the head-stage. Heading direction was binned into 3° bins and smoothed with a moving average filter with a width of 15°.

A notable electrophysiological parameter that may be important for odometry is the theta oscillation, a 7–11 Hz oscillation seen in the local field potential (LFP). The nature of the linear relationship between running speed and theta frequency was assessed by following methods used by (Jeewajee et al., 2008). To compensate for the fact that the animal’s position was sampled at 50 Hz and the LFP signal at 250 Hz, a 251-tap Blackman windowed band-pass *sinc* (sine cardinal) filter in the theta range (7–11 Hz) was applied to the LFP signal. By using the Hilbert transform the analytic signal was then determined and the instantaneous theta frequency calculated as the difference in phase between each time point, and then averaged over five consecutive values so that corresponding speed and EEG theta frequency could then be compared at 50 Hz. In order to quantify the speed–frequency relationship in each compartment, the *r* correlation coefficient was initially measured and transformed into a *z* correlation value with the Fisher transformation. A regression line was then fitted to the data from each recording session, and also for each individual run lasting more than 10 s in which running speed remained in the 4–30 cm/s range on both compartments. For each recording session, two values were then calculated for each compartment: (a) the intercept of the regression line and (b) the slope of the regression line.

For each run in both compartments, the sequence of the LFP detected in each recording session was zero-padded to the next highest power of 2 depending on the length of the sequence, and the power spectra constructed by using the fast Fourier transform (FFT) with the square-modulus of each Fourier frequency coefficient being the signal power at that frequency. A Gaussian kernel of width 2.0 Hz and SD 0.19 Hz was applied to produce a smoothed power spectrum. The mean theta frequency for each compartment was then defined as the frequency at which the power spectrum showed the maximum peak within the theta range (7–11 Hz).

#### Single Unit Firing Pattern Analysis

Single units were initially isolated using a cluster cutting program (Tint, Axona) in combination with KlustaKwik (Kadir et al., 2014) Automatic clustering of the data using KlustaKwik was fine-tuned and cleaned up manually (mainly to merge clusters falsely identified as separate).

One criterion for inclusion in the data set was that the single units had to each only be included once and so care was taken to make sure that co-recorded cells were unique. Initially the cluster space was examined to make sure that the single units were occupying distinct parts of the cluster space. Second, if two clusters were proximal the path/spike plots and firing rate-maps were examined to ensure that the firing fields were distinct. Additionally, temporal cross-correlograms were constructed and inspected to make sure that there was no refractory period. All putative single units were also tested for their grid score (see below), which needed to be above 0 for inclusion in the remaining analysis.

The firing pattern of grid cells was analyzed using standard methods for spatial cells (**Figure 3**), beginning with construction of a firing rate-map, and then analysis of this map for the several parameters detailed below. Following transformation of the position information (**Figure 3A**) the data were split into the two compartments (**Figure 3B**) and a rate-map was constructed for each one (**Figure 3C**). To accomplish this, for each rate-map the compartment under consideration was divided into 3 cm × 3 cm bins and the number of spikes emitted in that bin was divided by the total amount of time spent there, to normalize for inhomogeneous spatial sampling. The resulting map was then smoothed with a Gaussian kernel with a size of 5 × 5 bins. A number of summary measures were extracted from the rate-maps. These measures were as follows:

(a) Peak rate; the value of the bin with the highest firing rate. For the model these were set by hand, to match those seen in the real data: given this peak-rate “clamping,” the model was then interrogated with regard to the mean firing rate on the tilted surface, for which a statistical comparison was then made.
(b) Mean rate; the total number of spikes divided by the total dwell time;
(c) Spatial information (bits/spike); a measure of the extent to which the firing of a cell can be used to infer the location of the animal (Skaggs et al., 1993), was calculated as:

(1) $$I(R|X)\approx \sum_{i}p(\vec{x_{i}})f(\vec{x_{i}})\;log_{2}(\frac{f(\vec{x_{i})}}{F})$$

Where p(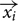) is the probability for the animal being at location 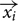, f(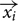) is the firing rate observed at 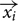 and *F* is the overall firing rate of the cell. For spatial information to be expressed in bits/spike the value obtained from Eq. 1 is divided by the overall mean firing rate.

(d) Coherence; the value of the correlation between the smoothed and unsmoothed firing rate-maps and is a measure of the ‘smoothness’ of the firing fields (e.g., round or smoothly elliptical firing fields will have a higher coherence score than irregular fields with jagged edges).
(e) Coverage; the percentage of bins with firing above a given percentage of the peak rate.
(f) Field number; this was calculated by performing a watershed segmentation on the rate-map and counting the number of distinctly labeled regions. The watershed algorithm operates by placing a ‘water source’ at each regional minimum in the inverse of the rate-map (so that local maxima are now local minima) and the entire ‘landscape’ is flooded from these sources and barriers are built when different water sources meet. The resulting set of barriers constitutes the segmentation of the image.
(g) Field size is the number of bins per field in which firing exceeded 50% of that field’s peak rate.
(h) Field center; the location of the peak bin.
(i) Inter-field distance; the mean of the distances between pairs of field centers (**Figure 3C**).

The next analyses were extracted from the spatial autocorrelogram SAC (Hafting et al., 2005; Sargolini et al., 2006; **Figure 3D**), generated by correlating the original rate-map with a copy of itself that was offset from the original at all possible *x*- and *y*-values. The resulting map emphasizes periodicities in the original image and is typically used to evaluate the metric properties and symmetry of grid cells. Symmetry of this plot was then quantified by applying a second autocorrelation procedure, detailed below.

Thus, from the SAC, we calculated the following:

(j) Grid field orientation; this was measured as the angle in degrees from an artificial horizontal at 3 o’clock on the SAC (dotted line in **Figure 3E**) moving anti-clockwise to the center of the nearest peak.
(k) Grid symmetry; this was measured by taking a circular section of the SAC centered on the central peak (which was subsequently excluded) and bounded by the edges of the peaks nearest the center, and then correlating rotated versions of the SAC with the original, producing a sinusoidal plot, or “symmetry curve,” of high and low correlation values. Positive peaks in this plot were defined as the regions where the derivative of the symmetry curve was zero and the second derivative negative. For a grid with hexagonal close-packed symmetry (the usual form in two dimensions), the symmetry plot would show peaks recurring every 60°; for a square arrangement these would be at 90°. Grid score was then calculated from the resulting plot by subtracting the smaller of the two values at 60 and 120 from the maximum of the values at 30, 90, and 150.

### Histology

At the end of the experiment the animals were deeply anesthetized and transcardially perfused with saline followed by paraformaldehyde. The brains were left in a 20% sucrose/4% paraformaldehyde solution for 24 h or until the brains had sunk to the bottom of the container. Forty micron sections were then cut on a cryostat, mounted on microscope slides, stained with cresyl violet, cover-slipped, and examined under a light microscope for electrode track placement.

## Results

### Model Analysis

**FIGURE 4:**
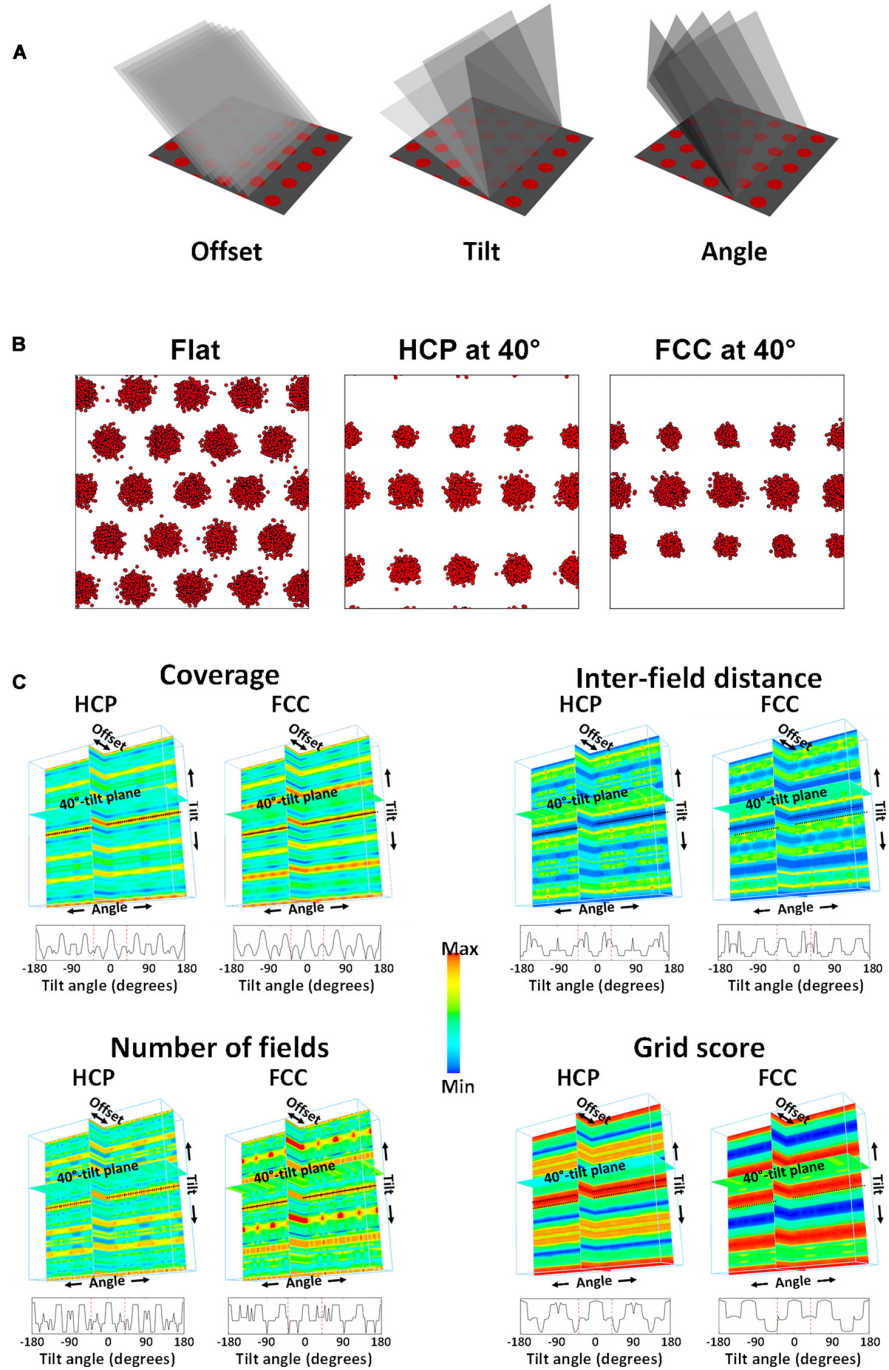
**Statistics extracted from the simulation. (A)** An imaginary plane was positioned in the HCP lattice (for which only its intersection with the horizontal plane is shown, for clarity) at various offsets (translations in the “*x*”-direction), tilts and rotations. **(B)** An example spikeplot from each of the lattices transected at 40° of tilt. Note the loss of hexagonal symmetry, and the decline in field number and size. **(C)** The parameters of interest were computed for every combination of offset, tilt, and angle. The final product is a four-dimensional (4D) plot, shown as a 3D solid with every voxel color-coded (color being the fourth dimension) to reflect the percentage coverage (as indicated by the scale bar). Shown here for four parameters are three orthogonal planar sections through this solid at zero offset, zero angle, and 40°-tilt (the tilt used in the recording experiment). The black dotted line indicates the horizontal plane (i.e., zero tilt). Since only tilt produced an appreciable variation in the parameters, the data are then collapsed across the irrelevant ones (offset and angle) and shown at the bottom of each panel as a line plot. The red dotted lines in this plot show the 40° point where the samples were drawn for analysis. Note that both on the 4D plots and on the line plots, the 40° point is far from the maximum seen on the horizontal plane.

As described in the Section “Materials and Methods,” model lattices (HCP and FCC) were generated and then transected by a square plane oriented at various angles in 3D space (**Figure 4A**). Simulated grid fields were then generated on the plane – an example for both HCP and FCC lattices is presented in **Figure 4B**. These spike plots were then converted into rate-maps and analyzed for their firing statistics in the same way as for the unit data.

Using the model, we generated predictions about how real grid cells would behave when recorded in a non-horizontal environment. **Figure 4C** summarizes the four most relevant measures, expressed as heat plots; these were the percentage of the simulated rate-map occupied with grid fields (“coverage”), the distances between the centers of the grid fields, the number of fields in the rate-map, and the grid score. For each of these parameters, a complete four-dimensional plot was generated, comprising three spatial axes for each of the spatial transformations (two rotations and a translation), with color for the fourth, summary measure. Because the resulting plot is a solid block we do not show it in its entirety, but have selected slices through it at pertinent places to show how the plots were generated and what the outcomes were (**Figure 4C**).

#### Visual Inspection of the Plots

Visual inspection of these plots reveals some details about the metric properties of the lattices. To begin with, it is evident that orientation angle and offset make little difference – there is little or no variation in the patterns along these axes, and so they will not be considered further. Tilt angle, by contrast, has a notable effect on the grid pattern for all the chosen parameters, as evidenced by the distinct periodicity in the tilt axis, resulting in a striking stripy pattern in the “backplane” (the tilt × angle plane). Inspection of the pattern of stripes reveals some of the symmetries of the two lattices. The HCP lattice displays mirror symmetry around the *x*-axis (the zero-tilt line, shown by the dotted black line in the plots) such that values either side of this are identical. By contrast, the FCC lattice does not possess this mirror symmetry due to the cyclic discontinuity that is present in the way the vertical layers of the lattice are stacked (ABCABC vs. the ABABAB layers of the HCP lattice). Instead, the symmetry line is offset with respect to the *x*-axis origin and lies at either -35° or +145°.

For both the HCP and FCC lattices, there is a clear peak (hottest colors) in the grid score, number of fields, and the amount of firing in the rate-map when the intersection plane was oriented horizontally (the zero-tilt line); this is unsurprising, because in the horizontal plane the fields are closest-packed. However, this closest packing never recurs in the HCP lattice (evidenced by the absence of recurrence of hottest colors in the patterns), while in FCC, there is a recurrence at -70° and +110°, such that the rate-maps generated from the plane/sphere intersections are identical to those when the plane is horizontal (not shown).

Most relevant for the present study is what happens when the intersection plane is tilted at 40°, which is the gradient of the sloped compartment in the single neuron experiment. In the heat plots, this is shown by the plane labeled “40° tilt plane” which shows the values of each of the four parameters, for HCP and FCC packings, at all orientations and offsets. For all four parameters the plane passes through a “trough” in the heat plot (evidenced by the cooler colors of these planes), such that predicted values would be considerably less than those of the horizontal tilt.

#### Quantitative Analysis of the Plots

We quantified the above observations by collapsing the 4D plots across orientation-angle and offset (since these had little effect), to produce average predicted values for all four parameters for the 40° tilt only. These were sampled at 10° intervals. The raw results are tabulated in **Table 1**, and the statistical comparisons presented below.

**Table 1:** Mean (±SEM) for the main parameters calculated from the grid cell data for the flat and sloped compartments.

	Model data					Neural data		
	Flat	Hexagonal close packed (HCP) slope	Change	Face centered cubic (FCC) slope	Change	Flat	Slope	Change
	**From the ratemaps**							
Peak rate (Hz)	38.44 (0.42)	37.06 (0.41)	-3.16%^∗^	37.67 (0.35)	-1.58%	9.54 (1.26)	8.61 (1.23)	-6.05%
Mean rate (Hz)	2.06 (0.01)	0.95 (0.01)	-53.97%^∗∗^	1.14 (0.01)	-44.46%^∗∗^	1.06 (0.19)	1.12 (0.22)	+8.53%^∗^
Spatial information (bits/spike)	2.42 (0.01)	3.53 (0.01)	+46.28%^∗∗^	3.26 (0.01)	+35.12%^∗∗^	0.57 (0.05)	0.49 (0.04)	-4.61%^∗∗^
Coherence	0.92 (0.00)	0.91 (0.00)	-0.43%^∗∗∗^	0.92 (0.00)	-0.18%^∗∗^	0.60 (0.02)	0.54 (0.02)	-8.77%^∗∗^
Coverage	29.83 (0.13)	13.78 (0.13)	-53.77%^∗∗∗^	16.57 (0.15)	-44.41%^∗∗∗^	19.26 (1.12)	23.22 (1.35)	+25.11%^∗∗^
Field number	21.67 (0.16)	11.28 (0.18)	-47.92%^∗∗∗^	13.31 (0.22)	-38 49%^∗∗∗^	4.73 (0.17)	5.25 (0.20)	+15.96% ^∗^
Field size (cm^2^)	137.78 (0.44)	123.17 (2.12)	-10.64%^∗∗∗^	125.12 (1.16)	-9.19%^∗∗∗^	235.61 (11.24)	221.14 (9.98)	-2.5%
Inter-field distance (cm)	23.38 (0.02)	25.20 (0.27)	+7.77%^∗∗∗^	23.53 (0.10)	+0.68%	15.78 (0.34)	14.59 (0.38)	-6.49%^∗^

	**From the spatial autocorrelograms (SAC)**							
Grid orientation (degree)	34.90 (2.95)	36.48 (4.38)	1.58°	38.39 (4.65)	3.50°	26.72 (2.25)	23.29 (2.23)	3.43°
Grid score	1.28 (0.00)	-0.14 (0.01)	-1.42^∗∗∗^	-0.04 (0.00)	-1.32^∗∗∗^	0.71 (0.07)	0.23 (0.08)	-0.48^∗∗∗^

**Symmetry peaks**	%	%		%		%	%	
1	0	0	0%	0	0%	0	2	+2%
2	0	0	0%	0	0%	5	7	+2%
3	100	0	-100%	0	-100%	93	68	-25%^∗∗^
4	0	66	+66%	0	0%	2	18	+16%^∗∗^
5	0	3	+3%	100	+100%	0	5	+5%^∗^
6	0	31	-31%	0	0	0	0	0%

*Significantly different at ^∗^*p* < 0.05; ^∗∗^*p* < 0.01; ^∗∗∗^*p* < 0.001.*

The peak firing rate of the plots changed only a few percent, as expected given that this was set by the spike-generating algorithm, but this was significant for HCP [*t*(35) = 2.19, *p* < 0.05] though not for FCC [*t*(35) = 1.31, NS]. The HCP effect is likely due to minor variance in the spike generation procedure and is not meaningful. However, the mean rate dropped to around half, which was a highly significant change [*t*(35) = 68.79, *p* < 0.0001 for HCP; *t*(35) = 44.66, *p* < 0.0001 for FCC]. This reflects the decreased number and size of fields (see below).

Spatial information (bits/spike) increased considerably for both lattices, which was highly significant [*t*(35) = 66.28, *p* < 0.0001 for HCP; *t*(35) = 51.05, *p* < 0.0001 for FCC], an effect that is due to the smaller fields (see below). Coherence changed minimally (<1%) which was expected due to the highly mechanistic nature of the simulation; the change was nevertheless statistically significant for both HCP [*t*(35) = 4.24, *p* < 0.0001] and FCC [*t*(35) = 3.82, *p* < 0.001].

Coverage (percentage of area bins in which there was firing) decreased to roughly half the area for both lattices, and was very highly significantly different [*t*(35) = 82.91, *p* < 0.0001 for HCP; *t*(35) = 60.62, *p* < 0.0001 for FCC] – this reflects the greater sampling of the inter-field spaces for the tilted plane relative to the flat plane. Likewise, the number of fields decreased considerably, by 40–50%, which was highly significant for both HCP [*t*(35) = 51.86, *p* < 0.0001] and FCC [*t*(35) = 31.58, *p* < 0.0001].

Field size also decreased slightly, by about 10%; this was significant both for HCP [*t*(35) = 7.40, *p* < 0.0001] and FCC [*t*(35) = 11.49, *p* < 0.0001]. Inter-field distance increased very slightly for HCP [by ∼8%; *t*(35) = 7.28, *p* < 0.0001] but not for FCC [*t*(35) = 1.64, NS].

We then subjected the transected firing field arrays to a 2D SAC procedure (Hafting et al., 2005; Sargolini et al., 2006), which enables extraction of metric properties of the field array of orientation and symmetry. Grid orientation changed hardly at all [*t*(35) = 0.33 for HCP, *t*(35) = 0.72 for FCC; both NS], which is to be expected since the basic lattice did not rotate. Grid score dropped notably, for both HCP [*t*(35) = 108.02, *p* < 0.0001] and FCC [*t*(35) = 434.16, *p* < 0.0001], reflecting the loss of hexagonal symmetry in the patterns. In support of this observation, while the number of symmetry peaks for the flat planes was 3 for 100% of the plots, reflecting the near-perfect hexagonal symmetry of the simulated lattices, this changed for HCP such that the majority of plots (66%) had four peaks while 3% had five and 33% had six. For FCC, all of the symmetry plots had five peaks.

These observations provide predictions for what would be observed in real grid cells recorded on a 40° slope, if the slope transected a lattice. We tested these predictions by recording grid cells as animals foraged on a two-part arena that was sloped in one half and flat in the other. Results from this experiment are described below.

### Positional Data and Its LFP Correlates

Before analyzing the neural data, we looked at the behavior in the apparatus to see if there were important differences between the flat and sloped compartments. We also looked at the LFP correlates of this behavior: we did this because evidence suggests that the dominant LFP rhythm in the entorhinal-hippocampal system, the theta rhythm, often shows a relationship to running speed (McFarland et al., 1975; Li et al., 2012).

**FIGURE 5:**
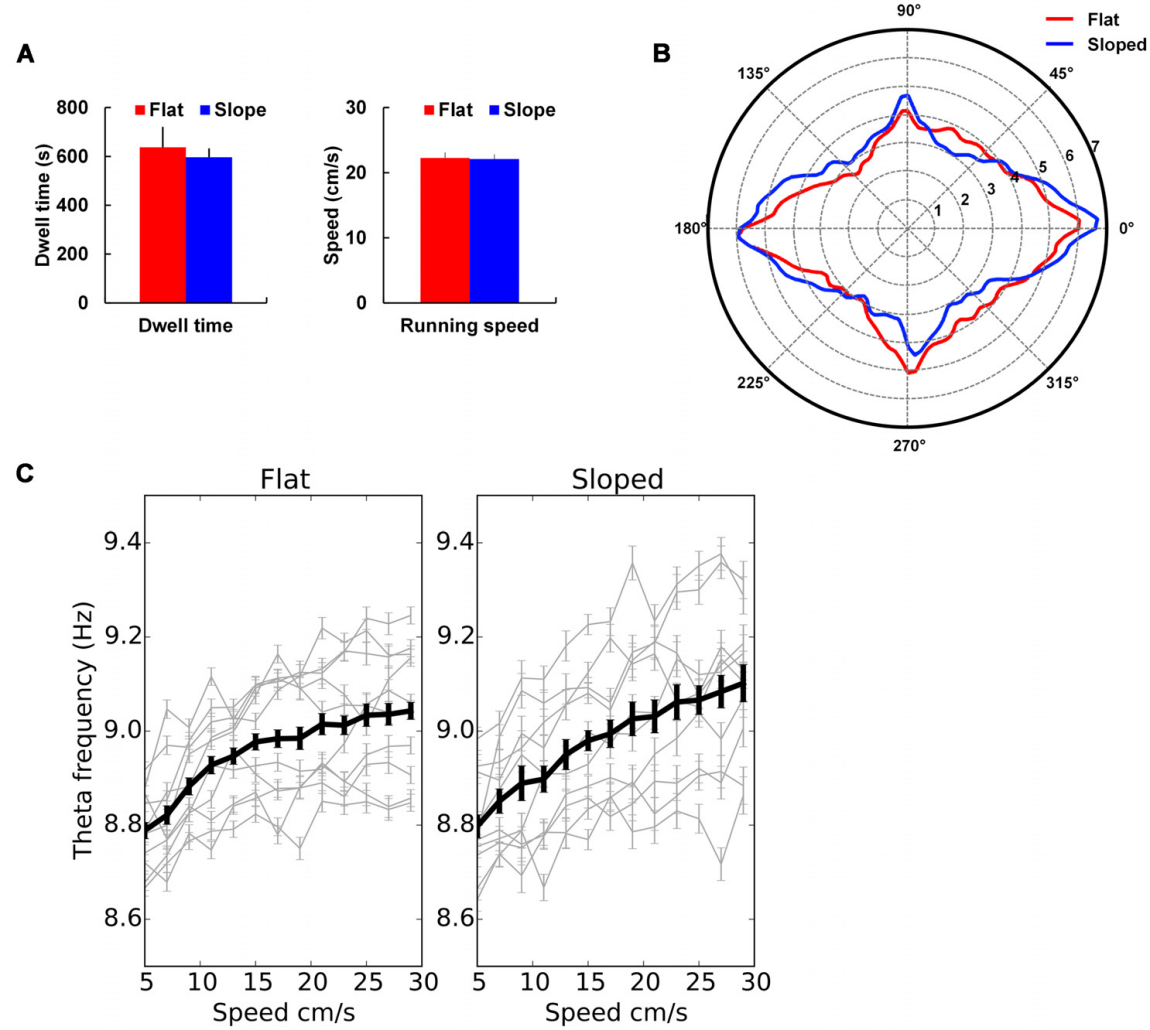
**Behavioral and local field potential (LFP) analyses. (A)** Mean dwell time and speed for the flat and sloped halves of the gradient box ± SEM. **(B)** Mean directional dwell time for the flat and sloped halves showing pronounced peaks aligned with the main axes of the recording environment, i.e., North–South and East–West. **(C)** Theta–speed relationship in both parts of the box showing no difference in theta–speed relationship between the two sections. Means ± SEM are shown with mean across all trials in thick black line and individual trials shown in light gray lines.

Locomotor behavior (**Figure 5**) was determined using the path data from the camera’s tracking of the LED on the animal’s head. After the position data had been transformed using the shrinking paradigm described in the Section “Materials and Methods,” the path of the animal across the recording trial was analyzed to extract speed, total dwell time, and distribution of running direction in the sloped half compared to the flat half of the arena.

There was no difference in the amount of time spent in the two compartments; rats spent on average 49.3 ± 3.4% of their time in the sloped compartment and 50.7 ± 3.4% in the flat compartment, which did not differ significantly [*t*(8) = -0.20, NS]. There was also no difference in running speed, this being 22.4 cm/s on the slope and 22.5 cm/s on the flat [*t*(8) = 0.91, NS].

There was a strong bias in directional heading in both compartments, but this was not different between the sloped and the flat sides. **Figure 5B** illustrates how the distribution of directional values is polarized along the length vs. width of the gradient box. An ellipse was fitted to these distributions and the major and minor axes of the ellipses calculated to quantify how the distributions were oriented. The major axis for the sloped and flat halves of the environment were oriented at 3.5° and 0.9°, respectively, showing a preference for the animals to run along the orienting axes of the gradient box. However, there was no difference in the probability distributions of the directional values in the sloped and flat parts of the environment when compared to a shuﬄed distribution of directional values (Kolmogorov–Smirnov test: *D* = 0.16, *p* > 0.05).

Using methods as reported in Jeewajee et al. (2008), we extracted theta oscillation (7–11 Hz) from the recorded LFP and then looked at whether it differed, in frequency, amplitude or relationship to running speed, between the sloped and flat compartments. In each recording session the mean theta frequency was determined as the frequency with the maximum peak in the EEG power spectrum filtered in the theta band (7–11 Hz) and across trials it averaged 8.84 ± 0.18 Hz on the slope and 9.19 ± 0.23 Hz on the flat which did not differ significantly [*t*(8) = 1.74, NS]. Similarly, the peak in the EEG power spectrum was compared across trials; it averaged 5.86 × 10^-7^ ± 2.0 × 10^-7^ WH^-1^ on the slope and 6.50 × 10^-7^ ± 2.3 × 10^-7^ WH^-1^ on the flat, which was again not significant [*t*(8) = 1.02, *p* > 0.05]. A linear relationship between running speed and theta frequency was evident for each trial for both compartments (**Figure 5C**). Comparisons for the speed–theta *z*-correlation value, intercept, and slope of the fitted regression line averaged across trials showed no difference between compartments (mean *z*-correlation was 0.09 ± 0.01 on the slope and 0.08 ± 0.01 on the flat, *t*(8) = -0.45, NS; mean intercept was 8.76 ± 0.04 Hz on the slope and 8.77 ± 0.03 on the flat, *t*(8) = 0.43, NS; mean slope was 0.01 ± 0.00 cm^-1^ on the slope and 0.01 ± 0.00 cm^-1^ on the flat, *t*(8) = -1.08, NS). All these analyses were then run again, this time averaging data from each rat and the comparisons showed again no differences between compartments.

Our conclusion from the behavior/LFP analysis is thus that there were no differences in behavioral or LFP parameters between the flat and sloped compartments.

### Single Neuron Firing Pattern Analysis

From the four rats, 76 cells were recorded in total and the data analyzed to determine which neurons met the criteria for being grid cells. Thirty were discarded due to low grid score and two due to being repetitions from previous days. In total 44 grid cells were analyzed from four animals across nine separate recording sessions, with one animal contributing to five separate recordings (number of cells per animal; *n* = 1, *n* = 38, *n* = 1, *n* = 4). An example of the firing of a single grid cell is shown in **Figure 3**, and the entire data set is shown in Supplementary Figure S1. Histology confirmed placement of electrodes in MEC or PaS.

**FIGURE 6:**
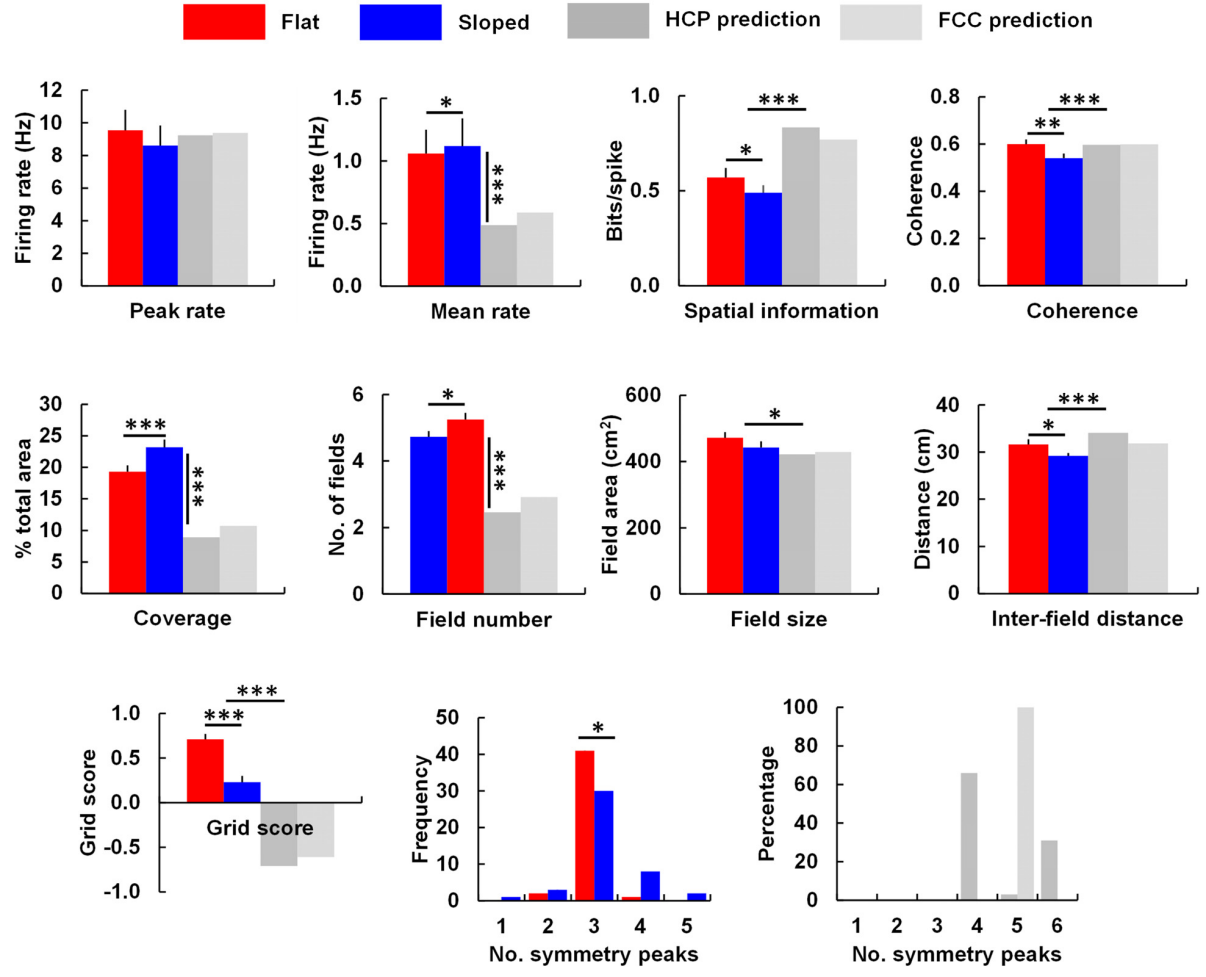
**The main grid cell firing parameters that differed between the flat and sloped compartments, compared against the predictions made by the HCP and FCC lattice models (see text and **Table 1** for details and statistical tests).** Note that peak rate for the model was set by hand to match the data; the important comparison therefore is what happened to the mean rate. Significantly different at ^∗^*p* < 0.05; ^∗∗^*p* < 0.01; ^∗∗∗^*p* < 0.001.

We analyzed the grid cell firing patterns as described in the Section “Materials and Methods.” The results are shown in **Figure 6** and listed in **Table 1**. Peak firing rates did not significantly change [*t*(43) = 1.59, *p* = 0.06], while the mean rate increased slightly [*t*(43) = 1.69, *p* < 0.05]. The mean rate change differed in sign from that predicted by the simulations: in the model, they *decreased*, to about half. A *t*-test comparing the percentage change from the HCP and FCC values combined against the neural data showed a very highly significant difference [*t*(114) = 18.86, *p* < 0.0001]. Thus, the firing rate in the sloped compartment was not what would be expected for a 40° transection of a close-packed grid field lattice.

There was significantly less spatial information content in the firing patterns on the slope [*t*(43) = -2.47, *p* < 0.05], an observation which contrasts with the predictions made by the model, in which spatial information increased markedly. In support of this, a *t*-test comparing the percentage change from the HCP and FCC values combined against the neural data showed a very highly significant difference [*t*(114) = 9.67, *p* < 0.0001].

As well as reduced spatial information content, the neural data also showed reduced spatial coherence [*t*(43) = -3.15, *p* < 0.01]; comparison with the HCP/FCC combined data found that this was a very much larger decrease than the minimal change predicted by the modeling [*t*(114) = 3.69, *p* < 0.0001]. There was an increased percentage of coverage (bins containing firing; [*t*(43) = 3.86, *p* < 0.001]). This is dramatically different from the predictions of the models, in which coverage was *reduced* by 50%; comparison of the neural data with the HCP and FCC data combined showed a highly significant difference [*t*(114) = 16.03, *p* < 0.0001]. There was also a significantly higher number of firing fields in the sloped half compared to the flat half [*t*(43) = 2.50, *p* < 0.05]; a change that, again, went in the opposite direction to that predicted by the model; comparison of the percentage change in the neural data with that in the HCP/FCC data combined showed a highly significant difference [*t*(114) = 12.220, *p* < 0.0001]. Fields were of similar size in both halves [*t*(43) = 1.21, NS]; the percentage change was different from that predicted by the models (comparison with HCP/FCC combined; *t*(114) = 2.23, *p* < 0.05), although it went in the same direction.

The increased coverage and field number, together with the reduced spatial information and coherence, is consistent with a general reduction in the spatial precision of firing on the slope, as can be seen by eye in the plots in the Supplementary S1.

A consequence of there being more fields in the sloped compartment compared to the flat, but the fields being the same overall size, was that they were closer together in the sloped compartment than in the flat [*t*(43) = 3.09, *p* < 0.05]. This contrasts with the findings in the simulated lattices in which inter-field spacing increased; the neural changes differed from the combined HCP/FCC changes significantly [*t*(144) = 5.17, *p* < 0.0001].

Analysis of the SAC revealed no significant difference in the orientation of the grid fields [*t*(49) = -1.56, *p* > 0.05]. Grid score declined significantly [*t*(35) = 5.27, *p* < 0.0001]; however, comparison with the predictions from the lattice (HCP and FCC combined) found that grid score in the neural data declined considerably less than that of the models [*t*(144) = 12.32, *p* < 0.0001]. The distribution of the number of symmetry peaks shifted between flat and sloped data: the number of occurrences of 1,2,3,4, and 5 symmetry peaks for the flat compartment were 0,2,41,1,0, respectively; for the sloped compartment the corresponding occurrences were 1,3,30,8, and 2 (note that three peaks corresponds to sixfold – i.e., hexagonal – symmetry). A chi-square test found these to be significantly different [*X*^2^(46) = 10.26, *p* = 0.04], indicating a slight decline in the hexagonal pattern, although far less than for the models (in which there was a complete loss of hexagonal – 3-peak – symmetry; **Table 1**).

### Histology

Histology (Supplementary Figure S2) showed that the grid cells were located in PaS (*n* = 1) and MEC (*n* = 3). The electrodes were likely in layers II–III (based on the tracks, and the lack of conjunctive cell properties).

## Discussion

We compared two hypotheses about how grid cells might organize their firing fields on sloping terrain (**Figure 1C**): (a) That their firing fields would adopt a pattern consistent with the slope having cut through a horizontally aligned, 3D, close-packed (**Figure 1D**), or (b) that their fields would have the same pattern that they show on a horizontal surface, as if the “grid” had simply tilted along with the (**Figure 1E**). We first modeled the predicted pattern if hypothesis (a) is the case, and found that at 40°, the slope would cut through a lattice of fields in such a way that the proportion of the surface covered by the fields would be considerably reduced, and there should be fewer fields and reduced hexagonal symmetry. We then tested these predictions by recording grid cells on a flat vs. sloped surface, and found that the lattice predictions were not upheld: there was not reduced coverage (coverage was, if anything, slightly increased) nor fewer fields (likewise), and there was only a slight decline in hexagonal symmetry, as measured by the grid score and number of peaks in the symmetry plots. Inspection of the firing rate-maps suggests that for the majority of cells, the pattern on the flat side simply flowed into the slope with little interruption (Supplementary S1), although this continuity was difficult to quantify. We conclude that hypothesis (b) has stronger support: it appears more likely that grid cells on a sloping surface form a 2D hexagonal close-packed array of the same form that they show on a horizontal surface. However, there were some differences in some of the firing statistics, particularly coverage, inter-field distances and grid score, that suggest that the distance-measuring functions of the cells were somewhat altered on the slope. Below, we discuss the predictions of the models, followed by the implications both of the preserved firing pattern and also of the alterations we saw, concluding with some speculations about how rats encode 3D space.

Our starting assumption for the modeling was that since grid cell firing fields form a hexagonal close-packed array on a flat surface, and then *if* they encode 3D space, they would similarly do so with a 3D close-packing. In other words, their firing fields would be spherical (instead of circular as they are on the horizontal plane) and packed together as efficiently as possible. There are two maximally efficient 3D close-packing structures (Gauss, 1831), known as HCP and FCC: these are subtly different but they do result in slightly different predictions for some situations, so we generated simulated firing fields in both forms. We then determined what would happen to the firing field statistics if these lattices were transected by finite-sized planes (of dimensions similar, with respect to the field size, to those of real-world recording environments) angled at various tilts with respect to the horizontal, and at various orientations and translations (“offsets”) with respect to a horizontal row of fields. Because the pattern seen with a horizontal transection is hexagonal close-packed, i.e., maximally efficient, tilts of the transection plane away from the horizontal always resulted in a decrease in packing density, which we called coverage, (**Figure 4**), though the maximum was regained periodically when the transection plane occasionally passed through another plane of close-packed spheres (e.g., at 70° tilt in the FCC lattice). Minimum coverage occurred for both lattices at all orientations and offsets at around 40°. At this tilt, which was also the tilt value chosen for the recording experiment, the average coverage would be around 50% of the horizontal value and the number of fields would be 40% of the horizontal value. We also examined the symmetry of the predicted patterns and found a decrease in the number of symmetry peaks.

The above analyses suggest that at a gradient of 40°, which is the maximum slope that our rats would readily explore, we should see large declines in several grid-cell firing parameters including field coverage, field number, inter-field distance, number of symmetry peaks, and grid score. We thus extracted the values for each of these parameters and compared them against the neuronal recording data, which were collected as the rats ran on either the horizontal surface or one tilted by degrees (**Figure 6**). Analysis of running speed and LFP found no significant differences between the sloped and flat compartments in either the behavioral parameters (running speed and distribution of running directions) or in the LFP, although there were slight non-significant decreases in theta frequency and amplitude. As mentioned earlier, the pattern of grid cell activity on the sloping surface was well preserved too; in contrast to the lattice-hypothesis prediction of reduced coverage and field number, we saw a preservation (and indeed, slight increase) in both of these parameters, consistent with alignment of the grid to the tilted surface.

Despite the preservation of firing pattern, on the sloping surface there was a slight fall in spatial information content and grid score, together with a decrease in the distance between field centers. Inspection of the firing rate-maps suggested a slight increase in background firing (see the data in Supplementary Figure S1, particularly cells for which the depth of modulation of the symmetry peaks is much less on the sloping side; cell r488_120124_t3_c3 being a good example). It is not clear why this degradation in the grid pattern occurred: one possibility is that the increased locomotor demands afforded by the steep slope reduced the attention the animals were able to pay to spatial cues. Since grid cells are known to use external landmarks such as boundaries to help anchor their firing (Barry et al., 2007; Stensola et al., 2015), this could result in less precise encoding of position by the cells, with increased extra-field firing and a consequent drop in grid score. Despite the decline in grid score, it remained significantly higher than that predicted by the models (**Figure 6**): also, the average number of peaks in the symmetry plots was not altered (**Figure 6**), confirming that the packing arrangement was not distorted on the slope.

These findings of preserved grid statistics lend support to our earlier proposal, detailed in Jeffery et al. (2013) and expanded in the companion paper (Jeffery et al., 2015), that the metric framework for the neural representation of space (the “cognitive map”) is 2D: that is, that grids form a close-packed 2D array that allows for distance-measuring on a surface, but not necessarily through volumetric space. Since rats do not generally move through volumetric space, the lack of spatial encoding in this dimension offers scant restriction to these animals’ spatial encoding efficiency. Indeed, a flat grid map offers some advantages – as shown by our modeling, a lattice produces reduced field packing density and grid symmetry for most planes other than horizontal and so a horizontally aligned and volumetrically organized grid lattice may actually be *less* useful to a surface-traveling animal. However, future experiments will be required to determine whether true movement through a volume – as might occur, for example, when moving through tree branches – elicits a volumetric close-packed grid structure even in rats.

The question arises as to the generality of these findings across other species, particularly humans. Although we ourselves are generally surface-traveling, our recent evolutionary ancestors were not, and so we may have evolved volumetrically organized grids. Studies of other volumetrically moving species such as bats and monkeys may help answer this question, until such time as it becomes possible to sample grids in human subjects too.

## Author Contributions

RH and KJ planned the experiment and analyses; RH generated and analyzed the lattice models, collected the single neuron data and analyzed the bulk of it. RH and KJ wrote the paper with discussion and input from GC and JW. GC contributed additional analyses.

## Conflict of Interest Statement

The authors declare that the research was conducted in the absence of any commercial or financial relationships that could be construed as a potential conflict of interest.
