# Supplementary material for: Grid cells on steeply sloping terrain: evidence for planar rather than volumetric encoding
Source: Front Psychol. 2015 Jul 15;6:925. doi: 10.3389/fpsyg.2015.00925 (PMC4502341; doi:10.3389/fpsyg.2015.00925)

# Supplementary Methods

## Model generation

Given a set of indices for the positions of the spheres $i$, $j$ and $k$ the x, y and z coordinates for the sphere, centres were calculated as follows for the HCP lattice:

|  | $\left[ \begin{matrix} 2i+(\left( j+k \right)mod2) \\ \surd3(j+ \frac{1}{3}\left( k mod2 \right) \\ \frac{2\surd6}{3}k \end{matrix} \right]r$ | (1) |
| --- | --- | --- |

Similarly, for the FCC lattice the coordinates of the sphere centres were calculated as:

|  | $\left[ \begin{matrix} 2i+j mod2+k mod3) \\ \surd3(j+ \frac{1}{3}\left( k mod3 \right) \\ \frac{2\surd6}{3}k \end{matrix} \right]r$ | (2) |
| --- | --- | --- |

A plane was then generated that intersected these sphere-packed volumes.

The plane was described by three non-colinear points, $p_{1}=(x_{1},y_{1},z_{1})$, $p_{2}=(x_{2},y_{2},z_{2})$ and $p_{3}=(x_{3},y_{3},z_{3})$, specifying vectors two vectors, $r0$ and $r1$ where:

|  | $r0= p_{2}-p_{1},$  $r1= p_{3}-p_{1}$ | (3) |
| --- | --- | --- |

Taking the cross-product of these two vectors gives the normal, $n$, to the plane where:

|  | $n=(a,b,c)$ | (4) |
| --- | --- | --- |

As the general form of the equation for the plane is:

|  | $Ax+By+Cz+D=0$ | (5) |
| --- | --- | --- |

Where,

|  | $D= -(ax_{1}+by_{1}+cz_{1})$ | (6) |
| --- | --- | --- |

When a plane intersects a sphere the resulting intersection is always a circle (cases where the plane hit the sphere at an exact tangent were ignored). The intersected circle coordinates, $x_{c}$, $y_{c}$and $z_{c}$were calculated according to the following:

|  | $\begin{matrix} x_{c}= x_{0}- \frac{A(Ax_{0}+By_{0}+Cz_{0}+D)}{A^{2}+B^{2}+C^{2}} \\ y_{c}= y_{0}- \frac{B(Ax_{0}+By_{0}+Cz_{0}+D)}{A^{2}+B^{2}+C^{2}} \\ z_{c}= z_{0}- \frac{C(Ax_{0}+By_{0}+Cz_{0}+D)}{A^{2}+B^{2}+C^{2}} \end{matrix}$ | (7) |
| --- | --- | --- |

Where$x_{0}$, $y_{0}$ and $z_{0}$ are the coordinates of the spheres making up either the FCC or HCP lattice specified in Equations 1 or 2 above. The equation for the intersected circle is given as:

|  | $r^{2}={(x-x_{c})}^{2}+{(y-y_{c})}^{2}+{(z-z_{c})}^{2}$ | (8) |
| --- | --- | --- |

The distance of the intersected circle centre to a sphere centre is:

|  | $d=\frac{\left\vert Ax_{0}+By_{0}+Cz_{0}+D \right\vert}{\sqrt{A^{2}+B^{2}+C^{2}}}$ | (9) |
| --- | --- | --- |

And the radius of an intersected circle is given by:

|  | $r=\sqrt{R^{2}-d^{2}}$ | (10) |
| --- | --- | --- |

Where R is the radius of the sphere. Given the above the conditions for the intersection of the plane with a sphere were $R>d$and, for no intersection, $R<d$ (conditions where $R=d$, i.e. sphere and plane tangency, were ignored). The packing constant (the fraction of space occupied by copies of a geometric body) for hexagonally packed circles^1^ is:

|  | $\frac{\pi}{\sqrt{12}}\approx0.90690$ | (11) |
| --- | --- | --- |

And for close-packed spheres is:

|  | $\frac{\pi}{3\sqrt{2}}\cong0.74048$ | (12) |
| --- | --- | --- |

Despite the receptive fields of grid cells being organised in a hexagonal close-packed array each field has an inhibitory surround where no spiking occurs which lowers the packing constant. Therefore to facilitate comparison with the experimental data the radius of the spheres was shrunk (toward the sphere centre) by some factor to better emulate this inhibition (i.e. $R$ in the above equations was reduced).

## Transformation of positional data

First, the four quadrangle vertices of the sloped half of the gradient box (as though it was laid down flat) were calculated as follows:

|  | $\begin{matrix} \left( {x1}_{dst},{y1}_{dst} \right),({x2}_{dst},{y2}_{dst})=\left( central_{x},central_{y}+50 \right), (central_{x}, central_{y}-50) \\ \left( {x3}_{dst},{y3}_{dst} \right),{(x4}_{dst},{y4}_{dst})=\left( central_{x},central_{y}+50 \right), (central_{x}, central_{y}-50) \end{matrix}$ | (13) |
| --- | --- | --- |

Where $central_{x}$and $central_{y}$are the x, y coordinates of the mid-point of the camera, $\left( {x1}_{dst},{y1}_{dst} \right)$is the top-right vertex of the flat half of the environment, $\left( {x2}_{dst},{y2}_{dst} \right)$is the bottom-right hand vertex, $\left( {x3}_{dst},{y3}_{dst} \right)$ is the top-left most vertex and $\left( {x4}_{dst},{y4}_{dst} \right)$is the bottom-left hand vertex. Units are in centimetres. These form the destination points for the transformation.

Second, the four actual vertices of the sloped half under the influence of the distortion, or source points, were determined empirically for marking the four vertices and measuring them via the camera and converting them from camera pixels to centimetres. If the four source vertices are called $\left( {x1}_{src},{y1}_{src} \right)$ to $\left( {x4}_{dst},{y4}_{dst} \right)$, the following system of linear equations was solved:

|  | $\left( \begin{matrix} {x1}_{src} & {x2}_{src} & {x3}_{src} \\ {y1}_{src} & {y2}_{src} & {y3}_{src} \\ 1 & 1 & 1 \end{matrix} \right).\left( \begin{matrix} \delta\\ \mu\\ \tau\end{matrix} \right)= \left( \begin{matrix} {x4}_{src} \\ {y4}_{src} \\ 1 \end{matrix} \right)$ | (14) |
| --- | --- | --- |

Homogeneous coordinates were extracted from the columns resulting from Equation 14 which were then scaled by the coefficients calculated in Equation 14:

|  | $A= \left( \begin{matrix} \delta. {x1}_{src} & \mu.{x2}_{src} & \tau.{x3}_{src} \\ \delta. {y1}_{src} & \mu. {y2}_{src} & \tau. {y3}_{src} \\ \delta& \mu& \tau\end{matrix} \right)$ | (15) |
| --- | --- | --- |

Third, these steps were repeated for the four sets of destination points, (${x1}_{dst}$, ${y1}_{dst}$) through (${x4}_{dst}$, ${y4}_{dst}$):

|  | $\left( \begin{matrix} {x1}_{dst} & {x2}_{dst} & {x3}_{dst} \\ {y1}_{dst} & {y2}_{dst} & {y3}_{dst} \\ 1 & 1 & 1 \end{matrix} \right).\left( \begin{matrix} \delta\\ \mu\\ \tau\end{matrix} \right)= \left( \begin{matrix} {x4}_{dst} \\ {y4}_{dst} \\ 1 \end{matrix} \right)$ | (16) |
| --- | --- | --- |

And,

|  | $B= \left( \begin{matrix} \delta. {x1}_{dst} & \mu.{x2}_{dst} & \tau.{x3}_{dst} \\ \delta. {y1}_{dst} & \mu. {y2}_{dst} & \tau. {y3}_{dst} \\ \delta& \mu& \tau\end{matrix} \right)$ | (17) |
| --- | --- | --- |
|  |  |  |

Fourth, the transformation matrix $C$ was specified as:

|  | $C=B . A^{-1}$ | (18) |
| --- | --- | --- |
|  |  |  |

Fifth, the homogeneous coordinates of each destination x, y point in the sloped half was calculated as the product of the transformation matrix, $C$, and the homogeneous coordinate of the source x, y point:

|  | $\left( \begin{matrix} x' \\ y' \\ z' \end{matrix} \right)=C.\left( \begin{matrix} x \\ y \\ 1 \end{matrix} \right)$ | (19) |
| --- | --- | --- |
|  |  |  |

Finally, the destination point was mapped back to the real plane via the homogeneous divide (i.e. normalized):

|  | $\begin{matrix} x'' & = & \frac{x'}{z'} \\ y'' & = & \frac{y'}{z'} \end{matrix}$ | (20) |
| --- | --- | --- |

Once the transformation matrix $C$ was found equations 19 and 20 were applied to each raw LED coordinate in the sloped half of the environment, replacing the original raw LED value.

# References

Chang, HC & Wang, LC (2010). A Simple Proof of Thue’s Theorem on Circle Packing. arXiv: 1009.4322v1

# Supplementary figures

**Supplementary Figure 1 a-d**

Summary plots for each grid cell, labelled with rat number (e.g., r486), recording date (yymmdd), tetrode, and cluster number. The top of each plot shows the combined spike plots with spikes (shown in red) overlaid on the animal’s path (gray line), rescaled on the sloped (left) sides as described in the Methods. Below left and right are the separate analyses for the sloped and flat sides respectively. Beneath the individual spike plots are rate maps with numbers showing peak firing rate (P), mean rate (M) and number of fields detected in the rate map (N). Below each rate map is the spatial autocorrelogram (SAC) with black lines radiating out from centre showing the angle/distance to the three peaks nearest the centre moving anti-clockwise from 3 o’clock. The colored central region is the circular mask encompassing these peaks used to generate the rotational correlation plot (the ‘symmetry’ plot) below the SAC. Each symmetry plot has local maxima (green squares) and minima (red squares). The number below the symmetry plots is the grid score for that cell (see main text for details of the calculation)

**Supplementary Figure 2**

Histological sections at low power (1-2x) and higher power (4.5x) from the four rats showing the estimated end-point of the electrode tracks (arrowheads) and the estimated anatomical region (MEC = medial entorhinal cortex; PaS = parasubiculum).

**Supplementary Figure 1 a**

**Supplementary Figure 1 b**

**Supplementary Figure 1 c**

**Supplementary Figure 1 d**

#

**Supplementary Figure 2**


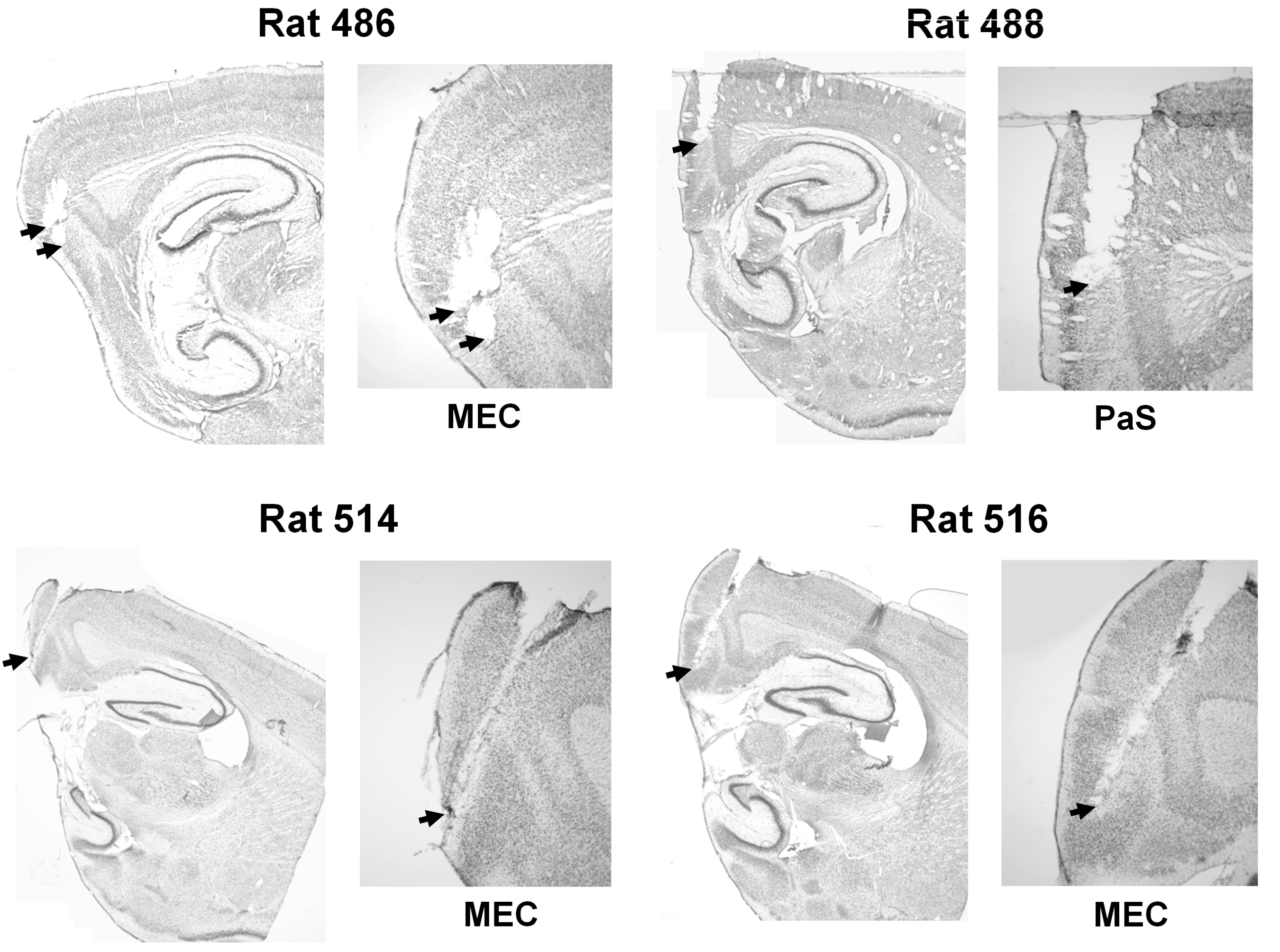

Supplement: Supplementary file 1 [file Data_Sheet_1.DOCX]
